# Supplementary material for: Susceptibility of Ovine Bone Marrow-Derived Mesenchymal Stem Cell Spheroids to Scrapie Prion Infection
Source: Animals (Basel). 2023 Mar 13;13(6):1043. doi: 10.3390/ani13061043 (PMC10044354; doi:10.3390/ani13061043)
Supplement: Supplementary file 1 [file animals-13-01043-s001.zip › animals-2184248-supplementary.pdf]

animals

MDPI

Supplementary

Table S1. Number of technical replicates used in each experiment.

Experiment		Technical replicates (*n*)	
oBM-MSC characterization	Adipogenic differentiation	Basal conditions	*n* = 3
		Differentiation conditions	*n* = 3
	Osteogenic differentiation	Basal conditions	*n* = 3
		Differentiation conditions	*n* = 3
	Chondrogenic differentiation	Basal conditions	*n* = 3
		Differentiation conditions	*n* = 3
Spheroid size measurement		Basal conditions	*n* = 4
Neurogenic differentiation	Nissl bodies staining (2D MSC)	Basal conditions	*n* = 3
		Differentiation conditions	*n* = 3
	Nissl bodies staining (MSC spheroids)	Basal conditions	*n* = 3
		Differentiation conditions	*n* = 3
	Expression analysis of neuronal markers (2D MSC)	Basal conditions	*n* = 2*
		Differentiation conditions	*n* = 2*
	Expression analysis of neuronal markers (MSC spheroids)	Basal conditions	*n* = 2*
		Differentiation conditions	*n* = 2*
ELISA (PrP^{Sc} detection) Times of study post-infection = 3	2D-MSCs	Basal conditions	*n* = 3#
		Neurogenic differentiation	*n* = 3#
	Spheroids	Basal conditions	*n* = 3#
		Neurogenic differentiation	*n* = 3#
Immunocytochemistry of spheroids (PrP^{Sc} detection)		Basal conditions	*n* = 4
		Neurogenic differentiation	*n* = 4
		Background control	*n* = 4
Cell viability assay	2D-MSCs	Basal conditions	*n* = 8$
		Neurogenic differentiation	*n* = 8$
	Spheroids	Basal conditions	*n* = 4$
		Neurogenic differentiation	*n* = 4$

* Each marker was analysed per triplicate in each technical replicate; # Technical replicates for each time of study (2, 5 and 8 dpi); $ Number of technical replicates per condition (non-innoculated, innoculated with control brain and inoculated with scrapie infected brain) and time (2, 5 and 8 dpi).
